# Inter-eye osmolarity differences in patients with symptomatic and non-symptomatic dry eyes

**DOI:** 10.5935/0004-2749.20200024

**Published:** 2020

**Authors:** Hugo Pena-Verdeal, Carlos García-Resúa, Covadonga Vazquez-Sanchez, Jacobo Garcia-Queiruga, María J. Giráldez, Eva Yebra-Pimentel

**Affiliations:** 1 Departamento de Física Aplicada (Area de Optometría), Universidade de Santiago de Compostela, Santiago de Compostela (Galicia), Spain

**Keywords:** Osmolar concentration, Dry eye syndromes, Lacrimal apparatus/chemistry, Tears/chemistry, Concentração osmolar, Síndromes do olho seco, Aparelho lacrimal/química, Lágrimas/química

## Abstract

**Purpose:**

To analyze whether inter-eye osmolarity differences were related to dry eye symptomatology.

**Methods:**

A total of 135 participants were randomly recruited from those who visited in the Optometry Clinic of the Optometry Faculty (Universidade de Santiago de Compostela). In a single scheduled session after the recruitment, Ocular Surface Disease Index was filled out following the standard instructions and TearLab measurements were made in both the participants’ eyes (10-15 min lapse). Osmolarity values were compared between the right and left eyes and the absolute inter-ocular difference (|OD-OS|) correlated with the Ocular Surface Disease Index score for the whole sample. Based on the Ocular Surface Disease Index score, the sample was divided into four symptomatic subgroups, and differences in the |OD-OS| values were calculated.

**Results:**

The whole sample showed a statistically significant inter-eye osmolarity difference (p=0.025; |OD-OS| = 9.2 ± 9.3 mOsm/l) and the correlation between Ocular Surface Disease Index and |OD-OS| (r=0.369; p<0.001). A statistically significant difference was found in the |OD-OS| value between symptomatic subgroups (Kruskal-Wallis, p=0.003). Mann-Whitney U test showed a significant difference between asymptomatic vs. moderate (p=0.006) vs. severe symp tomatic patients (p=0.001) and between mild vs. severe symptomatic patients (p=0.045), whereas no difference on |OD-OS| was found between participants with contiguous symptomatic subgroups (all p≥0.174).

**Conclusion:**

Tear film inter-eye osmolarity differences are significantly higher in severe dry eye disease symptoms.

## Abstract (pt)

**Objetivo:**

Analisar se as diferenças entre osmolaridade entre os olhos foram relacionadas à sintomatologia do olho seco.

**Métodos:**

Um total de 135 participantes foram recrutados aleatoriamente entre os indivíduos da Clínica de Optometria da Faculdade de Optometria (Universidade de Santiago de Compostela). Em uma única sessão agendada após o recrutamento, o Índice de Doenças da Superfície Ocular foi preenchido seguindo as instruções padrão e as mensurações do TearLab foram feitas em ambos os olhos dos participantes (lapso de 10 a 15 min). Os valores de osmolaridade foram com parados entre os olhos direito e o esquerdo e a diferença absoluta ocular (|OD-OS|) correlacionada com a pontuação do Índice de Doença da Superfície Ocular para toda a amostra. Com base na pontuação do Índice de Doença da Superfície Ocular, a amostra foi dividida em quatro subgrupos sintomáticos, e as diferenças nos |OD-OS| os valores foram calculad os.

**Re sultados:**

A amostra total mostrou uma diferença de osmolaridad e entre os olhos estatisticamente significativa (p=0,025; |OD-OS| = 9,2 ± 9,3 mOsm/l) e a correlação entre o Índice de Doença da Superfície Ocular e |OD-OS| (r=0,369; p<0,001). Diferença estatisticamente significativa foi encontrada no valor |OD-OS| entre os subgrupos sintomáticos (Kruskal-Wallis, p=0,003). O teste U de Mann-Whitney mostrou uma diferença significativa entre pacientes assintomáticos versus moderados (p=0,006) versus sintomáticos graves (p=0,001) e entre pacientes sintomáticos leves e graves (p=0,045), enquanto que nenhuma dif erença de |OD-OS| foi encontrada entre os participantes de subgrupos sintomáticos contíguos (todos p≥0,174).

**Conclu são:**

As diferenças entre osmolaridade inter-ocular do filme lacrimal são significativamente maiores nos sintomas graves da doença do olho seco.

## INTRODUCTION

Dry eye disease (DED) is typically considered as a symptomatic disease and is commonly diagnosed and graded based on symptomatology^(1-3)^. One of the commonly used methods for the symptomatic assessment is questionnaires, the Ocular Surface Disease Index (OSDI) as the principal method used as a diagnostic criterion^(4-6)^. An OSDI questionnaire is a self-administered questionnaire designed for immediate assessment of ocular surface symptoms related to chronic DED, their severity and effects on the patients’ daily life^(4-6)^. However, several studies have shown that symptoms were only weakly correlated with objective signs of dry eye^(7-9)^. This might be explained by the statistical independence of dry eye tests, which implies that these parameters were not used in the stratification of healthy/DED groups, which will become randomly distributed^(10)^. Therefore, the lack of correlation among DED tests (both signs and symptoms) was usually reported^(1)^.

Regarding the DED physiopathology, evaporationin duced tear hyperosmolarity is considered as the core mechanism and hallmark of the disease^(11-13)^. Tear hy perosmolarity is assumed to trigger a cascade of signaling events within the surface epithelial cells, which leads to the release of inflammatory mediators and proteases^(3,14)^. Therefore, the measurement of tear film osmolarity offers a valid information on the tear film status and has been proposed as the single best metric to diagnose and classify DED and a possible gold standard for the diagnosis of dry eye^(11-13,15,16)^. In clinical practice, the osmolarity test showed better accuracy than any other single tests to diagnose dry eyes and was the least variable sign^(10,13)^. For the DED diagnosis, dry eye tests are noted to be affected by temporal variability, which can negatively affect cross-section studies^(10)^. Tear osmolarity exhibits very little changes neither overtime nor between eyes in the healthy tear film; however, as the body loses control in the disease, tear film instability is reflected in steadily increasing eye-to-eye changes. While repeated measurements over a period of time have been shown to be low and stable in normal participants, those with DED were relatively increased and with unstable readings^(9,17,18)^. Indeed, the osmolarity widely varied or its increasing variation is a statistical characteristic of DED patients because of heteroscedasticity that might be considered as a clinical indication of tear film homeostasis loss occurring with dry eyes^(10)^. In addition, inter-eye variability has been found as a hallmark of DED, suggesting that the higher osmolarity of the two eyes could be used in clinical practice because the lower value seems to reflect transient effects in compensatory mechanisms^(1,15,16)^.

Although these inter-eye differences in tear osmolarity have been observed to diagnose DED, previous reports have not found its direct relationship with the symptomatology of dry eye^(1,15,16)^. Due to the diagnostic potential of heteroscedasticity, this study aimed to determine whether inter-eye osmolarity differences are re lated to DED symptomatology.

## METHODS

### 1.0 Sample size calculation and participants

To calculate the sample size, PS Power and Sample Size Calculations software Version 3.1.2 (Copyright^©^ by William D. Dupont and Walton D. Plummer) was used. The study was planned as a continuous response variable from participants evaluated according to their symptomatology status (OSDI score)^(1,4-6)^. Previous OSDI data indicate that the mean standard deviation (SD) of repeated measures is normally distributed with a value of 6.7^(1,4)^. To determine the minimal clinical difference proposed in the literature of 4.5 OSDI scores^(1,5)^, the minimum number of participants required is 72, in order to enable rejection of the null hypothesis with a probability (power) of 0.80. Therefore, the type I error probability associated with this test is 0.05. To achieve a more reliable study, a larger population was recruited. A total of 135 volunteer participants were randomly recruited among patients presenting to the Optometry Clinic of the Optometry Faculty (USC) for a regular eye examination (56 men and 79 women with a mean ± SD age of 49.7 ± 11.2 [range, 20-76] years). Prior to the inclusion in the final study group, those who had history of conjunctival, scleral, or corneal disease; prior eye surgery; glaucoma; diabetes mellitus; thyroid disorders, wearing contact lenses, was under any type of medication, or used artificial tears at the time of the testing session were excluded. All participants were in Spanish origin (from the Galicia region) and from a wide range of incomes and occupations. All participants provided their written informed consent to be included in the study. The study protocol adhered to the tenets of the Declaration of Helsinki and was approved by the Ethics Committee in the USC.

### 2.0 Procedure and environmental conditions

Only 135 participants who met the inclusion criteria of the study were scheduled for another visit for OSDI and tear osmolarity measurements. To minimize diurnal variation, all study sessions were done at the same time of the day (between 4.00 and 6.00 pm). Environmental conditions of the clinic and laboratory were always controlled and maintained under similar lighting, temperature (20-23°C), and humidity (50-60%) conditions.

#### 2.1 OSDI

First, upon arrival, participants were allowed to rest for 5-10 min to adapt to the laboratory conditions prior to osmolarity measurements, and then they are asked to complete an OSDI questionnaire^(1,4-6)^. To obtain comparable data between participants, questions were asked with reference to a 1-week recall period following the standardized interview model^(19)^. Responses were annotated by the interviewer for the subsequent numerical evaluation according to the published guidelines on a scale of 0 to 100, with higher scores representing greater disability^(1,4-6)^.

The OSDI questionnaire was administered in the scheduled study session and was considered to evaluate the existence and impact of dry eye symptomatology in participants. These OSDI scores were used to create subgroups for cluster analysis: asymptomatic (OSDI score of <13), mildly symptomatic (OSDI score between 13 and 23), moderately symptomatic (OSDI score between 23 and 33), and severely symptomatic (OSDI score of ≥33) participants^(1,4-6)^.

#### 2.2 Osmolarity measurement

In the second scheduled study session, tear film osmolarity was measured using the TearLab^TM^ osmometer (TearLab, San Diego, CA, USA)^(9,16,20)^. Quality control electronic check card was used daily to verify the correct status of the system (if reading was 334 ± 3 mOsm/l, the pen was working correctly). In all procedures, the same test card Lot number was used. The instrument and test cards used were kept in the same humidity and temperature-controlled room where the study was performed.

The first eye to be measured was randomly selected. Participants were seated with the chin tilted upward and eyes directed toward the ceiling. The instrument probe (housing the disposable microchip) was placed on the lower tear meniscus until a beep is emitted indicating the tear sample (0.05 µl) has been collected through capillary action. The TearLab converts the electrical impedance of the sample into osmolarity (mOsm/l), as displayed on the device screen (measurement range, 275-400 mOsm/l). The contralateral eye was measured after a 10-15 min interval to avoid inter-eye interference following the same protocol^(9)^.

### 3.0 Statistical analysis

SPSS statistical software v.19.0 for Windows (SPSS Inc., Chicago, IL) was used for data analysis. Significance was set at p≤0.05 for all statistical tests.

Prior to the analysis, the normal distribution of data was evaluated using the Kolmogorov-Smirnov test^(21)^. Osmolarity was normally distributed (p>0.05), whereas OSDI scores were non-continuously distributed (p<0.05). The inter-eye osmolarity differences were cal culated as the absolute difference between values obtained from both eyes of participants (|OD-OS|)^(16)^. Since the absolute inter-eye osmolarity difference was non-continuously distributed (Kolmogorov-Smirnov test: p<0.05), its differences between subgroups were assessed using the Mann-Whitney U and the Kruskal-Wallis was used to analyze differences between various subgroups. The correlation between eye osmolarities was calculated using the Pearson correlation, whereas Spearman Correlation was used when OSDI scores or absolute inter-eye difference parameters were used. Correlations were categorized as weak (0.2-0.4), moderate (0.4-0.6), substantial (0.61-0.8), and almost perfect (0.8-1.0).

## RESULTS

In the entire group, OSDI had median value of 22.9, with an interquartile range from 12.5 to 37.5. Tear osmolarity (Mean ± SD) was 316.1 ± 16.1 mOsm/l for the OD and 318.5 ± 17.3 mOsm/l for the OS. The difference in osmolarity between both eyes (p=0.025) was signi ficant, with an absolute inter-eye difference (Mean ± SD) of 9.2 ± 9.3 mOsm/l. A positive substantial significant correlation in osmolarity (Pearson correlation: r=0.704; p<0.001) was observed between the par ticipant’s both eyes.

**Figure 1:**
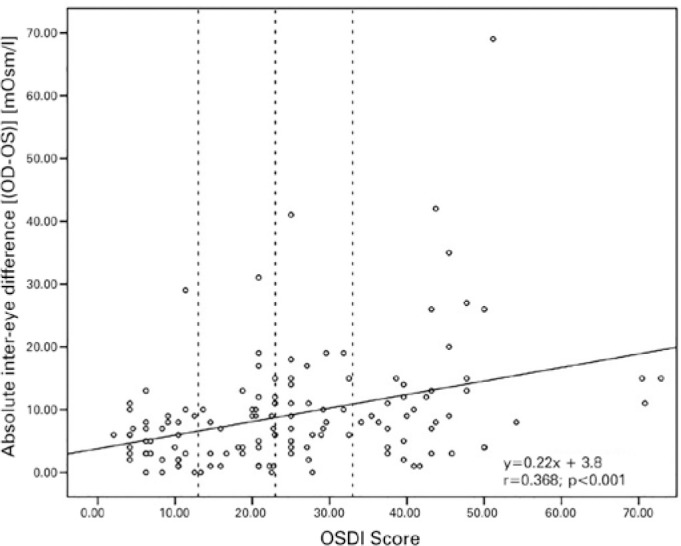
The absolute difference between the inter-eye tear osmolarity [OD-OS] and OSDI score in all samples. Non-symptomatic, mildly, moderately, and severely symptomatic groups are divided according to the vertical dashed lines. n=135.

Correlation analysis showed that the OSDI score was significantly positively but weakly correlated with the absolute inter-eye difference between eyes (Spearman correlation, |OD-OS| vs. OSDI score: r=0.369; p<0.001) (Figure 1).

When all 135 participants were grouped according to the OSDI score, 35 were found to be asymptomatic (25.92% OSDI of <13), 32 were mildly symptomatic (23.70% OSDI 13-23), 32 were moderately symptomatic (23.70% OSDI 23-33), and 36 were severely symptomatic (26.66% OSDI of ≥33). Table 1 shows the descriptive statistics and |OD-OS| in each symptomatic subgroup. The difference in |OD-OS| was statistically significant between subgroups (Kruskal-Wallis: p=0.003).

**Table 1:** Descriptive statistics and differences in the absolute inter-eye difference in osmolarity between participants grouped according to OSDI

		Group characteristics	Absolute inter-eyedifference (|OD-OS|)	
**Subgroup according to the OSDI score**	**n**	**Age [years] (Mean ± SD) OSDI [score] (Median)**	**Mean ± SD [mOsm/l]**	p-value^*^
Asymptomatic	35	48.9 ± 10.3 6.8	5.8 ± 5.2	0.003
Mildly symptomatic	32	49.8 ± 12.1 20.6	7.8 ± 6.6	
Moderately symptomatic	32	54.1 ± 9.1 27.1	9.8 ± 7.8	
Severely symptomatic	36	49.1 ± 11.8 43.2	13.3 ± 13.4	

SD= standard deviation;

* = Kruskal-Wallis test.

Mann-Whitney U test showed a significant difference between asymptomatic and moderately (p=0.006) and severely symptomatic (p=0.001) patients and between mildly and severely symptomatic patients (p=0.045). Nevertheless, no difference was found in the |OD-OS| value between contiguous symptomatic groups (asymptomatic vs. mildly symptomatic: p=0.174; mildly vs. mo derately symptomatic: p=0.229; and moderately vs. severely symptomatic: p=0.435).

## DISCUSSION

This study analyzed the inter-eye difference in osmolarity value and its relationship with DED symptoms. The OSDI score was positively but weakly correlated with the absolute inter-eye osmolarity difference (Figure 1). In addition, the difference in |OD-OS| was also statistically significant between the four symptomatic subgroups (Table 1). Previous studies have also measured the inter-eye osmolarity difference^(9,16,17,20,22-28)^; however, most of them have calculated this difference as a tangential analysis to the main objective of their study. In general, most studies also concluded that no osmolarity difference was observed between eyes in normal eyes as opposed to the pathological ones.

In this study, asymptomatic participants showed a re latively high mean osmolarity. This could be due to the presence of participants in the randomly selected sample with mild dry eyes who were misclassified as asymptomatic participants^(1,2,10)^. Hyperosmolarity affects the nerve function and morphology, and therefore, some participants classified as asymptomatic could be affected by this neuropathy^(3)^.

Tear film osmolarity is considered one of the core mechanisms of DED along with the tear film stability^(11-13,15,16)^. The inter-eye variability of the test was also greater in DED than in healthy patients, a characteristic that increases with disease severity and has been recommended as a feature that clinicians should specifically be looking upon diagnosis^(1,15,16)^. Sullivan advocated that between-eye differences beyond the threshold of 8 mOsm/l should be considered an indication of the tear film homeostasis loss that occurs with dry eye disease^(10)^. In this study, the absolute inter-eye osmolarity difference found in the whole sample was near to this value (|OD-OS| = 9.2 ± 9.3 mOsm/l) and was even higher for those with more severe symptoms (Table 1). Based on these results, Lemp et al.^(16)^ found that inter-eye osmolarity difference was correlated to disease severity and suggested that large osmolarity differences between eyes reflect an increase in disease severity, rather than an error in the test measurement. The same hypothesis was proposed by Potvin et al.^(29)^, who established that variability in tear osmolarity can also be a diagnostic indicator, where varied inter-eye measurements appear to increase with the dry eye severity. In addition to this hypothesis, osmolarity measurements in healthy patients seem to show a strong correlation without difference in the value between eyes^(20,25)^. On the contrary, patients with other dry eye-related pathologies, such as pterygium, also showed inter-eye differences: osmolarity in eyes with pterygium was significantly higher than those in the control (fellow) eye of the same patient^(26)^.

It should be noted that despite to the general trend of data, a group of participants still showed high inter-eye osmolarity differences and low symptomatology (Figure 1, left side) and vice versa (Figure 1, right down corner). Based on a previous report on the relatively high osmolarity mean found in asymptomatic patients, DED was associated with various manifestations, such as non-obvious disease involving ocular surface signs without rela ted symptoms (including neurotrophic conditions with the presence of dysfunctional sensation) and those with symptoms but without demonstrable ocular surface signs (including neuropathic pain)^(1,2,10)^. In addition, another source of error or limitation in this study was the use of one questionnaire only or indicator of the symptomatic status. Despite the fact that many other questionnaires have been established with concurrent validity against the OSDI in recent publications^(1)^, this questionnaire has limited number of questions. Moreover, because OSDI is a good screening tool for diagnosis, OSDI scores are not a competent indicator of a differential diagnosis in these types of dry eye; therefore, an additional diagnostic evaluation method (such as ocular surface staining, corneal estesiometry, etc.) is required to differentiate these asymptomatic groups. Future studies may also focus on analyzing other inter-eye parameters such as corneal or conjunctival damage or tear film volume and stability and its relationship with patient’s dry eye complaints or diagnosis.

Osmolarity difference between a patient’s eyes could be considered as one diagnostic parameter related to symptomatic complaints of dry eyes. These inter-eye differences could be related with symptoms than the absolute value of osmolarity as normal participants exhibit a very tight band of values within the homeostatic range, whereas participants with dry eye frequently exceed the healthy range^(9)^. In contrast, osmolarity differences between eyes have already been proposed, in terms of recorded tear osmolarity variability, which could be attributed to rightor left-handed operators who were more comfortable collecting tear samples from the left eyes. Therefore, they would achieve a more consistent and adequate position of the test card tip^(20)^. In this study, all osmolarity recordings were performed by the same investigator (left-handed), who was highly trained in performing TearLab measurements to prevent interobserver variance in the collection process. Keech et al.^(9)^ have reported that four consecutive measurements, whether at 15 or 1 min intervals, could be performed without significantly influencing the osmolarity values in participants with dry and normal eyes^(9)^. They also found a gradual increase between successive measures observed in the dry eye group using a 1-min time interval. Finally, they recommended collecting no more than four samples from a given eye with at least 60-s intervals between measurements to minimize the influence on values^(9)^. In our study, a 10-15-min interval was initiated, and only two osmolarity measurements were obtained (one per eye). This time interval should prevent inter-eye interactions.

In summary, this study showed that tear film inter-eye osmolarity differences are significantly higher in patients with severe DED symptoms.

OD= right eye (*oculus dexter*); OS= left eye (*oculus sinister*).

## References

[1] Wolffsohn JS, Arita R, Chalmers R, Djalilian A, Dogru M, Dumbleton K (2017). TFOS DEWS II Diagnostic Methodology report. Ocul Surf.

[2] Craig JP, Nichols KK, Akpek EK, Caffery B, Dua HS, Joo CK (2017). TFOS DEWS II Definition and Classification Report. Ocul Surf.

[3] Bron AJ, de Paiva CS, Chauhan SK, Bonini S, Gabison EE, Jain S (2017). TFOS DEWS II pathophysiology report. Ocul Surf.

[4] Schiffman RM, Christianson MD, Jacobsen G, Hirsch JD, Reis BL. (2000). Reliability and validity of the Ocular Surface Disease
Index. Arch Ophthalmol.

[5] Miller KL, Walt JG, Mink DR, Satram-Hoang S, Wilson SE, Perry HD (2010). Minimal clinically important difference for the ocular surface
disease index. Arch Ophthalmol.

[6] Patel VD, Watanabe JH, Strauss JA, Dubey AT. (2011). Work productivity loss in patients with dry eye disease: An
online survey. Curr Med Res Opin.

[7] Nichols KK, Nichols JJ, Mitchell GL. (2004). The lack of association between signs and symptoms in patients
with dry eye disease. Cornea.

[8] Schein OD, Tielsch JM, Munõz B, Bandeen-Roche K, West S. (1997). Relation between signs and symptoms of dry eye in the elderly. A
population-based perspective. Ophthalmology.

[9] Keech A, Senchyna M, Jones L. (2013). Impact of time between collection and collection method on human
tear fluid osmolarity. Curr Eye Res.

[10] Sullivan B. (2014). Challenges in using signs and symptoms to evaluate new biomarkers
of dry eye disease. Ocul Surf.

[11] Liu H, Begley C, Chen M, Bradley A, Bonanno J, McNamara NA (2009). A link between tear instability and hyperosmolarity in dry
eye. Invest Ophthalmol Vis Sci.

[12] Stahl U, Willcox M, Stapleton F. (2012). Osmolality and tear film dynamics. Clin Exp Optom.

[13] Tomlinson A, Khanal S, Ramaesh K, Diaper C, McFadyen A. (2006). Tear film osmolarity: determination of a referent for dry eye
diagnosis. Invest Ophthalmol Vis Sci.

[14] Schargus M, Ivanova S, Kakkassery V, Dick HB, Joachim S. (2015). Correlation of tear film osmolarity and 2 different MMP-9 tests
with common dry eye tests in a cohort of non-dry eye
patients. Cornea.

[15] Jacobi C, Jacobi A, Kruse FE, Cursiefen C. (2011). Tear film osmolarity measurements in dry eye disease using
electrical impedance technol ogy. Cornea.

[16] Lemp MA, Bron AJ, Baudouin C, Benitez Del Castillo JM, Geffen D, Tauber J (2011). Tear osmolarity in the diagnosis and management of dry eye
disease. Am J Ophthalmol.

[17] Nolfi J, Caffery B. (2017). Randomized comparison of in vivo performance of two point-of-care
tear film osmometers. Clin Ophthalmol.

[18] Gokhale M, Stahl U, Jalbert I. (2013). In situ osmometry: validation and effect of sample collection
technique. Optom Vis Sci.

[19] Fowler FJ. (1995). Improving survey questions: design and evaluation.

[20] Szczesna-Iskander DH. (2016). Measurement variability of the TearLab osmolarity
system. cont lens anterior eye.

[21] Armstrong RA, Davies LN, Dunne MC, Gilmartin B. (2011). Statistical guidelines for clinical studies of human
vision. Ophthalmic Physiol Opt.

[22] Dimit R, Miller W, Picus A, Leach N, Bergmanson J. (2010). Diurnal osmolarity in silicone hydrogel contact lens
wearers. Optom Vis Sci.

[23] Khanal S, Millar TJ. (2012). Barriers to clinical uptake of tear osmolarity
measurements. Br J Ophthalmol.

[24] Montani G. (2013). Intrasubject tear osmolarity changes with two different types of
eyedrops. Optom Vis Sci.

[25] Caffery B, Chalmers RL, Marsden H, Nixon G, Watanabe R, Harrison W (2014). Correlation of tear osmolarity and dry eye symptoms in convention
attendees. Optom Vis Sci.

[26] Ozsutcu M, Arslan B, Erdur SK, Gulkilik G, Kocabora SM, Muftuoglu O. (2014). Tear osmolarity and tear film parameters in patients with
unilateral pterygium. Cornea.

[27] Aslan Bayhan S, Bayhan HA, Muhafız E, Bekdemir Ş, Gürdal C. (2015). Effects of osmoprotective eye drops on tear osmolarity in contact
lens wearers. Can J Ophthalmol.

[28] García N, Melvi G, Pinto-Fraga J, Calonge M, Maldonado MJ, González-García MJ. (2016). Lack of agreement among electrical impedance and freezing-point
osmometers. Optom Vis Sci.

[29] Potvin R, Makari S, Rapuano CJ. (2015). Tear film osmolarity and dry eye disease: A review of the
literature. Clin Ophthalmol.

